# High-Performance Oil Well Cement with Modified Calcium Carbonate Whiskers: Enhancing Durability Under HTHP Conditions

**DOI:** 10.3390/ma18051021

**Published:** 2025-02-26

**Authors:** Xingguo Liu, Jiankun Qin, Rongdong Dai, Hanguo Zhou, Xueyu Pang, Xuhui Chen

**Affiliations:** 1 Research Institute of Petroleum Engineering Technology, Shengli Oilfield, Dongying 257000, China; liuxingguo582.slyt@sinopec.com (X.L.); dairongdong.slyt@sinopec.com (R.D.); chenxh359.slyt@sinopec.com (X.C.); 2 Postdoctoral Scientific Research Working Station of Shengli Oilfield, SINOPEC, Dongying 257000, China; 3 Shengli Oilfield, China Petroleum and Chemical Corporation, Dongying 257000, China; zhouhanguo.slyt@sinopec.com; 4 School of Petroleum Engineering, China University of Petroleum (East China), Qingdao 266580, China; x.pang@upc.edu.cn; 5 Key Laboratory of Unconventional Oil & Gas Development, China University of Petroleum (East China), Ministry of Education, Qingdao 266580, China

**Keywords:** calcium carbonate whiskers, toughening agents, rheological properties, cement durability, high-temperature and high-pressure (HTHP) conditions

## Abstract

This study investigates the effect of incorporating modified calcium carbonate whiskers, treated with tetraethyl orthosilicate (TEOS), to enhance the mechanical properties and sealing integrity of oil well cement under high-temperature and high-pressure (HTHP) conditions. Traditional cement systems are prone to brittleness and cracking under dynamic loads, leading to compromised wellbore sealing performance. Our findings demonstrate that fiber-toughened cement slurry improves the toughness and sealing performance of the cement annulus, maintaining gas tightness and mechanical integrity under cyclic alternating pressures at 150 °C. Specifically, the inclusion of 5% modified whisker fibers improves compressive strength by 24.5% and flexural strength by 43.3% while maintaining stable rheological and thickening properties. These results support the hypothesis that modified whisker fibers enhance the durability and sealing integrity of cement wellbores under extreme conditions, providing a practical solution for challenging cementing applications.

## 1. Introduction

Cementing is a vital phase in oil and gas well drilling and completion operations, as its quality directly determines the lifespan and productivity of wells, serving as a cornerstone technology for efficient hydrocarbon development [1]. During this process, cement slurry solidifies between the casing and the borehole wall, forming a cement sheath that provides structural support and protection for the casing while achieving effective zonal isolation. This function is essential for the subsequent extraction of oil and gas [2,3]. However, sidetracking in mature wells, an important approach to revitalizing low-yield, inefficient reservoirs, often encounters challenges due to the small borehole diameter and narrow annular space. These limitations compromise the quality of the cement sheath and the integrity of the wellbore [4].

The situation is further complicated under high-temperature and high-pressure (HPHT) conditions, where cement strength typically undergoes significant deterioration due to strength retrogression. In this process, the amorphous hydration products of cement, such as C-S-H, crystallize at temperatures above 110 °C, weakening the material. This degradation is particularly evident in silica-enriched cement systems, which, despite stabilizing the cement at lower temperatures, still experience considerable loss of strength when exposed to elevated temperatures above 150 °C [5]. At 200 °C, strength retrogression can lead to up to an 80% reduction in compressive strength and a significant increase in permeability due to phase transformations, such as the formation of tobermorite and xonolite [6,7]. To mitigate this effect, various toughening agents, including latex fibers, silica flour, and nanoparticles like nano-graphene, have been incorporated into cement to enhance its mechanical properties and resistance to degradation. Silica flour, in particular, has been shown to improve short-term strength stability, while nanoparticles offer better long-term enhancement of Young’s modulus [8]. Nevertheless, while these materials can delay strength retrogression, they do not completely eliminate it, suggesting that further research is necessary to optimize cement compositions for HPHT environments.

Additionally, traditional cement sheaths, characterized by high brittleness and limited deformability, are prone to cracking and detachment from the casing or borehole wall when subjected to dynamic loads or vibrations, such as perforation, fracturing, and acidizing. These issues prevent long-term effective zonal isolation, particularly in narrow annular cementing, where thinner cement sheaths and lower displacement efficiency exacerbate the problem. Such deficiencies not only undermine cementing quality but also reduce the operational lifespan of wells and, in severe cases, render them unusable [9].

To address the brittleness of cement sheaths, the introduction of toughening materials has become a widely used approach [10]. By incorporating these materials into cement slurry, the flexural strength, deformability, and sealing performance of the cement stone can be enhanced, improving its ability to withstand complex operational conditions. Recent advancements have explored various toughening techniques and modified materials with notable results. For example, Zhao et al. demonstrated that sulfonate-modified latex powder (SISBR) enhances the flexural strength of cement and reduces its elastic modulus. With an optimal addition of 2.4%, the 7-day compressive strength of the cement stone reaches 41.5 MPa, the flexural strength reaches 7.24 MPa, and the elastic modulus decreases to 3.16 GPa [11]. Zhai’s research on surface-modified rubber powder showed that a 15% addition improved the 28-day compressive strength of cement to 42.4 MPa and the flexural strength to 6.0 MPa [12].

Similarly, Cheng explored the toughening effect of ultrafine microsphere rubber powder (MRP) and identified an optimal addition of 0.5%, resulting in a 7-day compressive strength of 28.6 MPa, a flexural strength of 12.4 MPa, and an elastic modulus of 3.97 GPa [13]. Gao optimized particle size distribution to design an elastic expansion slurry system, achieving tensile strength, compressive strength, elastic modulus, and Poisson’s ratio of 38.6 MPa, 4.25 MPa, 3.62 GPa, and 0.37, respectively, under 85 °C and 50 MPa conditions [14]. Patel proposed a triazine-based polymer (TzP), which reduced the elastic modulus of cement to 11.0 GPa after three days of curing at 82 °C and 20 MPa while maintaining high compressive strength [15]. Ahmed investigated the use of perlite particles, which effectively reduced the elastic modulus and brittleness of cement under high-temperature conditions, achieving a modulus of 15.1 GPa after 24 h of curing at 150 °C and 20.7 MPa [16].

Globally, leading oilfield service companies such as Baker Hughes and Schlumberger have developed advanced toughened cement slurry systems that have been successfully applied in complex operational environments. For instance, Baker Hughes’ DuraSetTM system incorporates toughened fibers and crosslinked polymers to enhance the toughness of cement sheaths, while Schlumberger’s FlexSTONE system integrates sulfurized rubber powder and inorganic fibers, achieving an exceptionally low elastic modulus (as low as 1.38 GPa). However, the high cost of these systems limits their widespread application in domestic markets [17]. In contrast, domestic research has primarily focused on the development of cost-effective toughening materials, such as modified polypropylene fibers and latex cement, which have demonstrated success in improving zonal isolation in high-pressure wells. Nonetheless, the performance of these materials still lags behind international standards, particularly in terms of elasticity [18,19,20,21,22,23].

Currently, the compatibility of toughening materials with cement slurry performance remains a significant challenge for narrow annular cementing applications. Traditional short fiber toughening techniques often encounter issues such as fiber agglomeration, leading to poor rheological properties of the slurry. Furthermore, limitations in mixing processes restrict fiber dosage, hindering large-scale applications. Whiskers, a novel type of inorganic fiber material, offer promising potential due to their small diameter, uniform cross-section, exceptional mechanical strength, and excellent thermal resistance [24,25,26]. However, conventional whiskers exhibit low surface activity, limiting their compatibility with cement slurry systems. To address this issue, tetraethyl orthosilicate (TEOS) is used to modify the whiskers by coating their surface with a nano-SiO_2_ layer, enhancing the activity of surface hydroxyl groups and improving interfacial bonding between the whiskers and the cement matrix. This modification is aimed at strengthening the mechanical properties and toughness of the cement stone. The effectiveness of this modification will be evaluated through experimental data, and the conclusions will discuss whether the modified whiskers enhance the durability and sealing integrity of the cement system, providing a viable solution for improving cementing performance under challenging conditions.

## 2. Raw Materials and Formulation Design

### 2.1. Raw Materials

The materials utilized in this study comprised Class G oil well cement (supplied by Swei, Weifang, China), calcium carbonate (CaCO_3_) whiskers (Kelong Chemical Reagent Factory, Chengdu, China), TEOS (Kelong Chemical Reagent Factory, Chengdu, China), ammonia solution (NH_3_·H_2_O, Kelong Chemical Reagent Factory, Chengdu, China), and laboratory tap water (local source). Additional additives (Chuanfeng Chemical Engineering Co., Ltd., Chengdu, China) included silica fume, a lattice expansion agent, a high-temperature fluid loss additive, a solid friction reducer, a high-temperature retarder, and a rheology modifier. These materials and instruments were meticulously selected to ensure accuracy and reliability in the experimental results.

### 2.2. Experimental Equipment

(1) WQF-520 Fourier Transform Infrared Spectrometer

The WQF-520 Fourier Transform Infrared (FTIR) Spectrometer (Nicolette Corporation, Long Beach, CA, USA) was used to identify functional groups and analyze molecular structures in the samples. The procedure involved grinding the samples into fine powder; mixing them with spectroscopically pure potassium bromide (KBr), a chemical compound commonly used as a pelletizing agent in infrared spectroscopy due to its transparency in the infrared region; and pressing the mixture into transparent pellets using a hydraulic press. The pellets were placed in the sample chamber, and FTIR spectra were recorded within the 400–4000 cm^−1^ range. The obtained spectra were analyzed to detect chemical bonds, evaluate structural characteristics, and confirm material composition.

(2) X Pert MPD PRO X-Ray Diffractometer

The X Pert MPD PRO X-Ray Diffractometer (Malvern Panalytical Company, Almelo, The Netherlands) was used to analyze the crystal structure and phase composition of the materials. Samples were prepared by uniformly spreading fine powders onto a flat glass slide or a zero-background holder. The instrument generated X-ray beams at a fixed wavelength, and diffraction patterns were collected across a 2θ angle range. The data were analyzed using software (Highscore 5.0) to identify crystal phases, determine lattice parameters, and quantify crystallinity. Proper calibration and alignment of the instrument were conducted before each analysis to ensure accuracy.

(3) Quanta 450 Environmental Scanning Electron Microscope (ESEM)

The Quanta 450 ESEM (FEI Corporation, Hillsboro, OR, USA) was used for high-resolution imaging and analysis of the microstructure and morphology of materials. Samples were prepared by mounting them on aluminum stubs with conductive adhesive and sputter coating with a thin gold or platinum layer to enhance conductivity. The microscope was operated under vacuum or low-pressure modes, depending on the sample’s moisture content. Images were captured at magnifications of 2000×, 2500×, 3000×, and 5000× to provide detailed views of the surface structure of the materials. Elemental analysis was performed using Energy-Dispersive X-ray Spectroscopy (EDS) (FEI Corporation, USA) attached to the ESEM. Calibration of magnifications and detector settings was conducted to maintain accuracy.

(4) Cement Ring Integrity Apparatus

**Figure 1:**
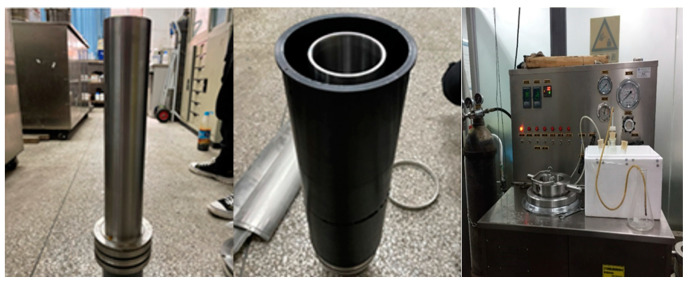
Cement ring integrity apparatus.

As shown in Figure 1, the cement ring integrity apparatus (Self developed equipment, China) is used to simulate the conditions encountered in the field, testing the sealing performance of cement rings under variable pressure. The process involves preparing the apparatus by installing the cement ring inside the device, followed by sealing it with a rubber casing. The cement ring is subjected to internal variable pressures, which simulate real-life conditions. The apparatus ensures the integrity of the cement ring’s seal through a series of steps, including setting the pressure and monitoring for any leakage. Finally, the results are observed to assess the sealing effectiveness, ensuring the cement ring maintains its structural integrity under stress.

(5) ZNN-D6 Six-Speed Rotational Viscometer

The ZNN-D6 viscometer (Haitongda Company, Qingdao, China) was used to evaluate the rheological properties of cement slurries by measuring their viscosity at different shear rates. The slurry was poured into the viscometer cup, and the rotating spindle was immersed. Measurements were taken at six predefined rotational speeds, and the data were used to calculate plastic viscosity and yield point. Proper calibration was conducted before each test to ensure consistent and accurate viscosity readings.

(6) TG-8040B High-Temperature and High-Pressure Thickening Instrument

The TG instrument (Model Setline STA, Caluire, France) was used to evaluate the thickening behavior of cement slurries under simulated downhole conditions. The slurry was prepared and poured into a test chamber, which was sealed to withstand high pressure and temperature. The instrument was programmed to gradually increase the temperature and pressure to match wellbore conditions. Consistency changes were recorded over time to determine the thickening time and setting behavior of the slurry under extreme conditions.

(7) NYL-300 Pressure Testing Machine

The NYL-300 pressure testing machine (Wuxi Building Materials, Instruments and Machinery Factory, Wuxi, China) was used to determine the compressive strength of hardened cement samples. Cylindrical samples were prepared, cured under controlled conditions, and mounted between the machine’s loading platens. Pressure was applied uniformly at a constant rate until the samples fractured. The maximum load at failure was recorded and used to calculate the compressive strength. Regular calibration of the machine was performed to ensure the accuracy of the results.

### 2.3. Experimental Method

#### 2.3.1. Pretreatment of Calcium Carbonate Whiskers

A 30% hydrogen peroxide solution (50 mL) was measured and transferred into a beaker and then diluted with deionized water to a total volume of 300 mL [27]. Calcium carbonate whiskers (15 g) were added to the solution. The beaker was placed in an ultrasonic cleaner, where the mixture was simultaneously stirred and ultrasonicated at room temperature for 15 min. After this process, the mixture was washed with deionized water, filtered under vacuum, and dried to obtain the pretreated calcium carbonate whiskers.

#### 2.3.2. Preparation of Modified Calcium Carbonate Whiskers

The pretreated calcium carbonate whiskers were mixed with ethanol and deionized water (using 400 mL of ethanol with a mass ratio of ethanol to deionized water of 2:1) in a beaker. A suitable amount of ammonia solution was added to adjust the pH of the mixture. The mixture was then transferred into a 1000 mL flask and stirred magnetically for 30 min.

Subsequently, a measured amount of TEOS was weighed and mixed with a small amount of ethanol. The resulting solution was added dropwise to the mixture over the course of 1 h. The reaction was allowed to proceed under continuous stirring at room temperature for a specified duration. The resulting product was washed thoroughly with deionized water, filtered, and dried in an oven at 60 °C for 24 h to yield the final modified calcium carbonate whiskers.

This two-step process ensured that the calcium carbonate whiskers were adequately pretreated and modified, improving their compatibility and performance in subsequent applications.

### 2.4. Preparation and Testing of Cement Slurry

The cement slurry was prepared and tested in accordance with the standards outlined in GB/T 19139-2012 (Test Methods for Oil Well Cement) [28], SY/T 5504-2008 (Evaluation Methods for Oil Well Cement Additives) [29] and SY/T 6466-2016 (Performance Test Methods for Oil Well Cement Stones) [30]. The formulation of the cement slurry consisted of Swei (Shengwei) cement as the base material, with 30% silica fume, x% composite elastic toughening agent, 1% lattice expansion agent, 2% high-temperature fluid loss additive, 2% solid friction reducer, 0.5% high-temperature retarder, 1% rheology modifier, and 46% mixing water. The water-to-cement ratio was maintained at 0.44. The mixing and testing conditions simulated downhole environments, with a circulating temperature of 120 °C and a static temperature of 150 °C. The properties of the cement slurry, including density, rheological behavior, and thickening performance, and compressive strength, flexural strength, and elastic modulus of the hardened cement stone, were systematically evaluated to ensure the accuracy and reliability of the results.

## 3. Results and Discussions

### 3.1. Characterization and Analysis of Modified Whiskers

#### 3.1.1. Infrared Spectroscopy Analysis

**Figure 2:**
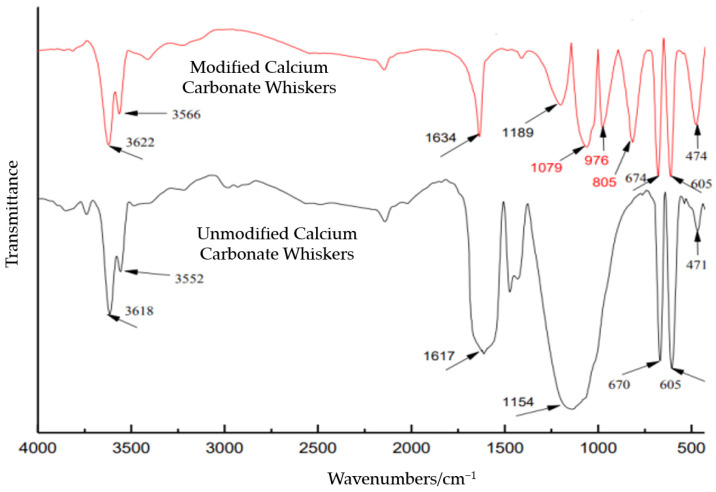
Infrared spectra of two types of calcium carbonate whiskers.

The infrared spectra of ordinary calcium carbonate whiskers and modified calcium carbonate whiskers are shown in Figure 2.

From Figure 2, it can be observed that ordinary calcium carbonate whiskers exhibit distinct characteristic peaks. The peaks at 3618 cm^−1^ and 3552 cm^−1^ correspond to the stretching vibration absorption of crystallization water and surface -OH groups, respectively. A bending vibration peak of crystallization water is observed at 1468 cm^−1^. The peak at 1617 cm^−1^ is attributed to the characteristic absorption of the active Ca^2+^ ions on the whisker surface. Additionally, the peak at 1154 cm^−1^ corresponds to the antisymmetric stretching vibration of tetrahedral SO_4_^2−^ groups, while peaks at 670 cm^−1^ and 605 cm^−1^ represent the asymmetric bending vibrations of SO_4_^2−^ groups. Lastly, a symmetric bending vibration peak of SO_4_^2−^ groups appears at 471 cm^−1^.

In the spectrum of modified calcium carbonate whiskers, in addition to the characteristic peaks of crystallization water and SO_4_^2−^ groups, new peaks appear at 1079 cm^−1^, 976 cm^−1^, and 805 cm^−1^, which are characteristic absorption peaks of SiO_2_. Specifically, the peak at 1079 cm^−1^ is associated with the antisymmetric stretching vibration of Si-O-Si bonds, the peak at 976 cm^−1^ corresponds to the bending vibration of Si-OH bonds, and the peak at 805 cm^−1^ represents the symmetric stretching vibration of Si-O bonds. The infrared spectrum clearly indicates the presence of SiO_2_ in the modified calcium carbonate whiskers, confirming successful modification.

#### 3.1.2. X-Ray Diffraction (XRD) Analysis

**Figure 3:**
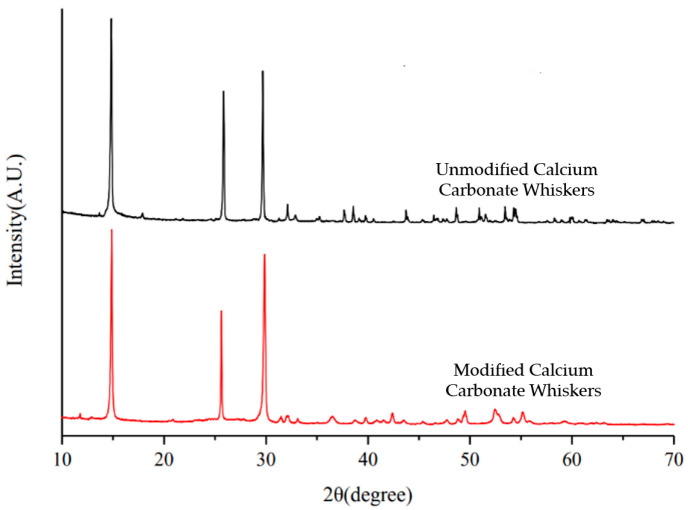
XRD characterization of calcium carbonate whiskers and modified calcium carbonate whiskers.

The XRD patterns of ordinary calcium carbonate whiskers and modified calcium carbonate whiskers are presented in Figure 3.

From Figure 3, it can be observed that the characteristic diffraction peaks of modified calcium carbonate whiskers closely align with those of ordinary calcium carbonate whiskers. Notably, no characteristic diffraction peaks of SiO_2_ are observed in the XRD pattern of the modified whiskers. This indicates that the SiO_2_ coating formed on the surface of the whiskers during the experiment is amorphous in nature.

#### 3.1.3. Surface Morphology Analysis of Whiskers

**Figure 4:**
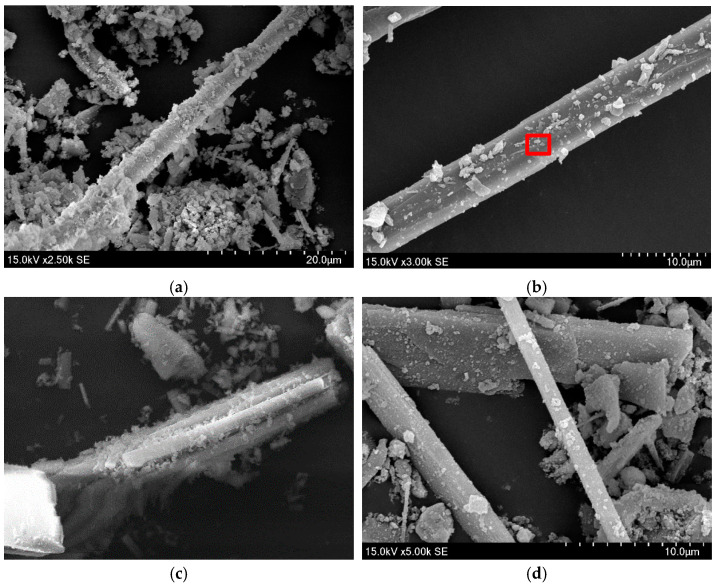
Surface morphology of modified whiskers. (**a**) 2500× magnification of the sample; (**b**) 3000× magnification of the sample; (**c**) 5000× magnification of the sample; (**d**) 5000× magnification of the sample.

**Figure 5:**
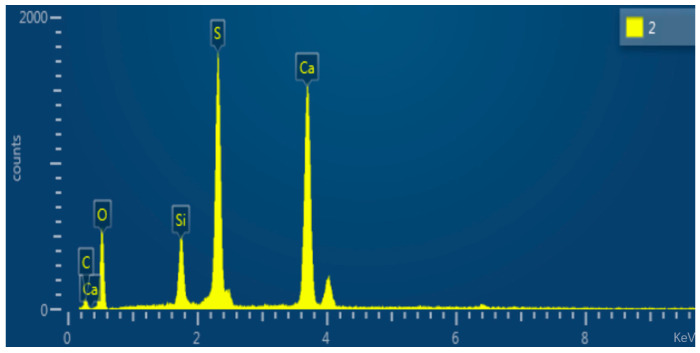
EDS elemental analysis of modified calcium carbonate whiskers.

A small amount of modified calcium carbonate whiskers was prepared for surface morphology analysis using a Scanning Electron Microscope (SEM). The SEM results are shown in Figure 4, and the red-marked regions in the images were subjected to Energy-Dispersive Spectroscopy (EDS) elemental analysis. The EDS results are displayed in Figure 5 and summarized in Table 1.

The SEM images of modified calcium carbonate whiskers reveal the presence of numerous rod-like whiskers with minimal agglomeration. This indicates that the modification process improved the dispersion performance of the whiskers. The improvement can be attributed to the higher surface charge density achieved after modification, which introduced strong negative charges on the whisker surfaces. This enhanced electrostatic repulsion between the whiskers, overcoming the hydrogen bonding forces that typically cause particle agglomeration. As a result, the modified whiskers exhibited excellent dispersion properties.

**Figure 6:**
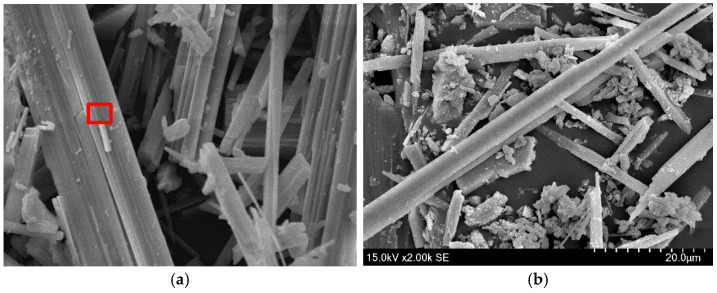
Surface morphology of unmodified whiskers. (**a**) 2000× magnification of the sample; (**b**) 2000× magnification of the sample.

**Figure 7:**
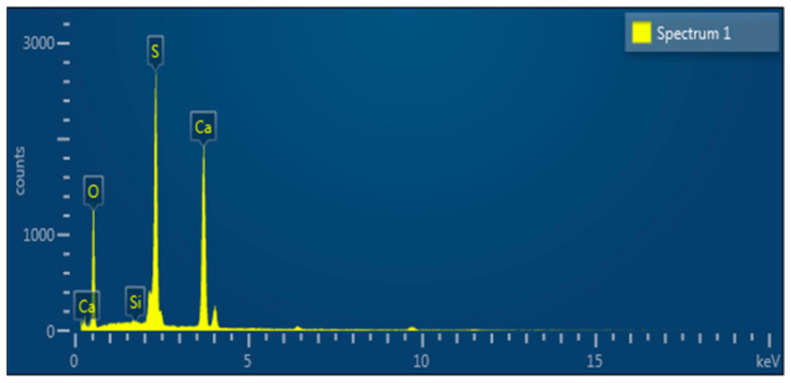
EDS elemental analysis of unmodified calcium carbonate whiskers.

To compare, SEM micrographs of unmodified calcium carbonate whiskers were also captured, as shown in Figure 6, and the EDS elemental analysis of the red-marked regions is shown in Figure 7 and Table 2.

The unmodified calcium carbonate whiskers exhibit smooth surfaces with almost no defects, while the modified whiskers show increased surface roughness. The SEM images of the modified whiskers clearly show numerous fine particles loaded onto the whisker surfaces, along with noticeable blocky and layered deposits. This indicates that SiO_2_ particles were successfully deposited on the whisker surfaces.

The EDS analysis further confirms this observation. The unmodified whiskers show no detectable Si element (Table 2), while the modified whiskers contain Si with a weight percentage of 5.91% (Table 1). The presence of Si is attributed to the hydrolysis of TEOS under alkaline conditions, which produced nanoscale SiO_2_ particles with strong adsorption and binding properties. These SiO_2_ nanoparticles adhered to the calcium carbonate whisker surfaces, forming blocky and layered adsorption layers. Thus, the above analysis confirms the successful modification of calcium carbonate whiskers using TEOS.

**Figure 8:**
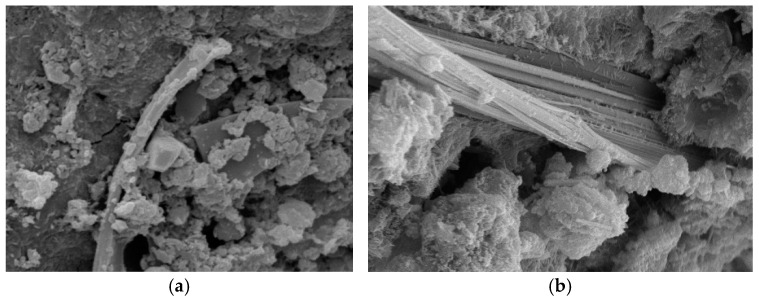
Modified whiskers embedded in the cement matrix. (**a**) 2000× magnification of the sample; (**b**) 5000× magnification of the sample.

Figure 8 shows the microstructure of cement stone containing modified calcium carbonate whiskers. It can be observed that the modified whiskers are tightly embedded within the hydration products of the cement matrix. The bonding strength between the whiskers and the cement stone has been enhanced. This improvement is attributed to the SiO_2_ particles loaded on the whisker surfaces, which increased the dispersion of the whiskers in the cement slurry and strengthened their interfacial bonding with the cement matrix. Consequently, the incorporation of modified whiskers has greatly improved the performance of the cement stone.

### 3.2. Study on Modified Whisker Fiber Formulation of Cement Stone Under 150 °C Curing Conditions

To determine the optimal dosage of modified whisker fibers in cement slurry, the mechanical properties of cement stone, particularly the improvement rate of flexural strength, were used as evaluation indicators. The optimal dosage of modified whisker fibers was identified, followed by an assessment of the cement slurry properties and mechanical performance of cement stone at this dosage.

#### 3.2.1. Effect of Modified Whisker Fiber Dosage

The mechanical properties of cement stone, including the 48 h flexural and compressive strengths, were used to determine the optimal dosage of modified whisker fibers. The dosage range for modified whisker fibers was selected based on the previous literature on fiber reinforcement in cement systems. A dosage range of 3%, 5%, and 7% by weight of cement was chosen to evaluate the effect of increasing fiber content on the mechanical properties of cement stone. Previous studies [27,31,32,33] have shown that fiber dosages within this range significantly enhance the strength and toughness of cement materials without negatively affecting the rheological properties of the slurry. The 3% dosage was selected as a lower bound to observe the initial effects of fiber reinforcement, while the 7% dosage was chosen to assess whether further increases in fiber content lead to diminishing returns in terms of mechanical improvement.

The cement slurry formulation is as follows: Shengwei cement + 30% silica fume + x% whisker fibers + 1% lattice expansion agent + 2% high-temperature fluid loss additive + 2% solid friction reducer + 0.5% high-temperature retarder + 1% rheology regulator + 46% water. The static temperature was maintained at 150 °C. The mechanical properties of cement stone at different modified whisker fiber dosages are shown in Table 3.

From Table 3, it can be observed that the 48 h flexural strength of cement stone exhibits a gradual increase with the addition of modified whisker fibers. When the dosage of modified whisker fibers reaches 5%, the compressive strength of the cement stone increases by 24.5% compared to the blank sample, while the flexural strength improves by 43.3%, demonstrating a toughening and strengthening effect. However, as the dosage increases to 7%, although the mechanical properties continue to improve, the 48 h compressive strength increases by only 2.5%, and the 48 h flexural strength increases by just 3.4% compared to the 5% dosage. The incremental improvement becomes marginal, and considering production costs, the optimal dosage of modified whisker fibers is determined to be 5%, where the mechanical performance of cement stone is enhanced while production costs remain reasonable.

#### 3.2.2. Performance of Cement Slurry with Optimal 5% Dosage of Modified Whisker Fibers

To investigate the impact of the optimal 5% dosage on cement slurry properties, the basic performance of cement slurry and the mechanical properties of cement stone were evaluated. The proportions of the cement slurry formulation were based on established guidelines for high-temperature cementing applications and the experimental conditions in this study. A base formulation consisting of Class G oil well cement, silica fume, and a series of additives was selected to ensure a balanced slurry with stable rheological properties, fluid loss control, and high-temperature resistance. The inclusion of 30% silica fume was chosen based on its proven ability to enhance the strength and durability of cement systems, particularly in high-pressure, high-temperature environments. The selected additives were included in proportions consistent with standards API 10B-2 [34] to optimize slurry performance under the experimental conditions of 150 °C. The water-to-cement ratio of 0.44 was chosen to maintain adequate slurry consistency while ensuring optimal thickening and setting behavior.

The cement slurry formulation is as follows: Shengwei cement + 30% silica fume + 5% modified calcium carbonate whisker fibers + 1% lattice expansion agent + 2% high-temperature fluid loss additive + 2% solid friction reducer + 0.5% high-temperature retarder + 1% rheology regulator + 46% water. The fundamental properties of modified whisker fiber cement slurry are shown in Table 4.

From Table 4, it can be seen that the basic performance of modified whisker fiber cement slurry is similar to that of unmodified whisker fiber cement slurry, indicating that the modification of whisker fibers does not adversely affect the slurry properties. The density of the modified whisker fiber cement slurry is 1.90 g/cm^3^, with a fluidity of 22 cm, zero free liquid, and an API fluid loss of 38 mL, reflecting excellent slurry performance.

**Figure 9:**
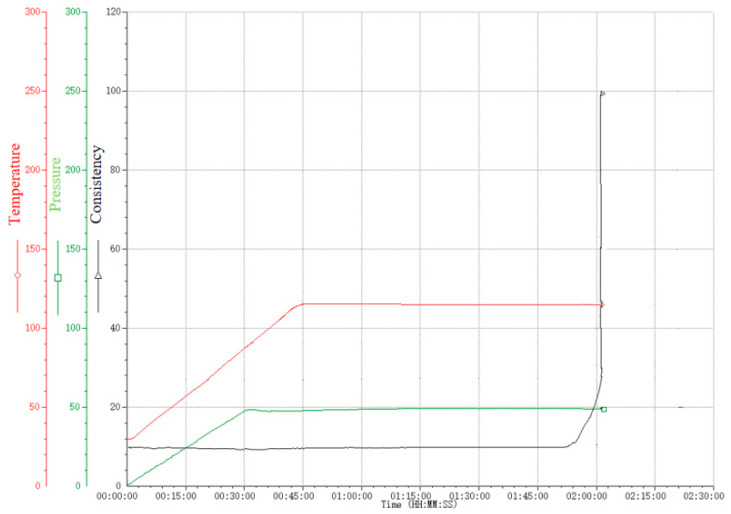
Thickening curve of cement slurry with different dosages of modified whiskers.

Figure 9 illustrates the thickening curve of the modified whisker fiber cement slurry. It shows that with a 5% dosage of modified whisker fibers, the initial thickening value is 14.5 Bc, and the thickening curve remains stable without undesirable phenomena such as “bulging” or “step changes”. The transition time for thickening is less than 30 min, indicating favorable thickening performance.

#### 3.2.3. Mechanical Properties of Cement Stone with the Optimal Dosage of Modified Whisker Fibers

The mechanical properties of cement stone with the optimal dosage of modified whisker fibers were tested. The cement slurry formulation is as follows: Shengwei cement + 30% silica fume + 5% modified calcium carbonate whisker fibers + 1% lattice expansion agent + 2% high-temperature fluid loss additive + 2% solid friction reducer + 0.5% high-temperature retarder + 1% rheology regulator + 46% water, with the test temperature maintained at 150 °C.

The 48 h mechanical performance evaluation results of modified whisker fiber cement stone are presented in Table 5**.**

**Figure 10:**
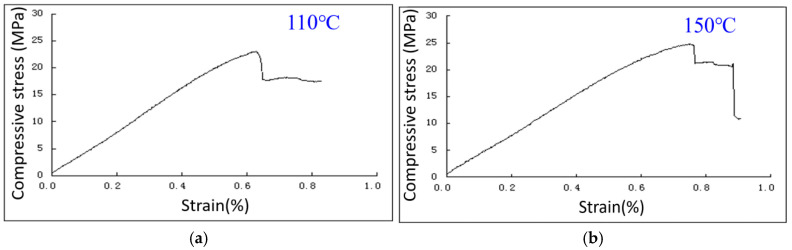
Stress–strain curve of composite resilient cement stone at 48 h. (**a**) Compressive stress–strain curves at 110 °C; (**b**) compressive stress–strain curves at 150 °C.

**Figure 11:**
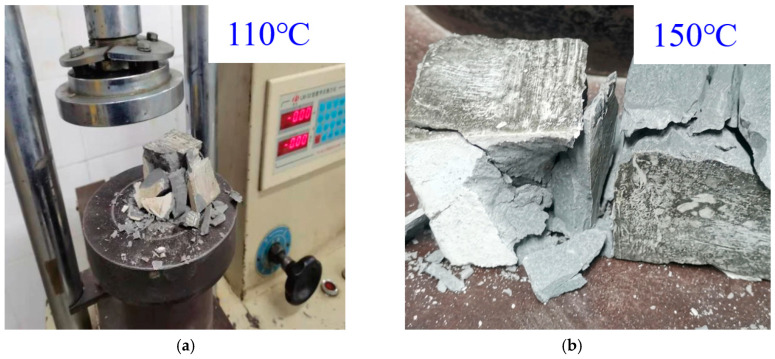
Compressive strength testing of composite resilient cement stone. (**a**) Compression fracture of samples cured at 110 °C; (**b**) compression fracture of samples cured at 150 °C.

The experimental data show that the mechanical properties of composite resilient cement stones are influenced by temperature and fiber modification. As shown in Figure 10, the 48 h stress–strain curves indicate that at 110 °C, the cement stone exhibits a peak compressive strength of approximately 28 MPa, with a gradual decline after the peak, demonstrating a brittle failure mode. At 150 °C, the peak compressive strength increases to around 30 MPa, and the post-peak drop becomes steeper, suggesting a more pronounced effect of high temperature on the failure mode. The failure images (Figure 11) further reveal that at 110 °C, the samples break into larger, sharper fragments, indicating typical brittle failure. In contrast, at 150 °C, the fragments are finer with more surface cracks, indicating more ductile failure behavior due to higher temperature effects. Furthermore, Table 5, comparing the mechanical properties, confirms the effect of fiber modification. The 48 h compressive strength and flexural strength of pure cement slurry are 28.5 MPa and 6.0 MPa, respectively. Adding unmodified whisker fibers increases these to 30.5 MPa and 7.0 MPa, while modified whisker fibers further enhance the performance, achieving 35.5 MPa compressive strength and 8.6 MPa flexural strength, with a 43.3% improvement in flexural strength compared to pure cement slurry. These results indicate that temperature and fiber modification play a crucial role in improving the mechanical properties and failure modes of composite resilient cement stones.

#### 3.2.4. The Role of Modified Whisker Fibers Toughening in the Integrity of Cement Annular Wellbores

**Figure 12:**
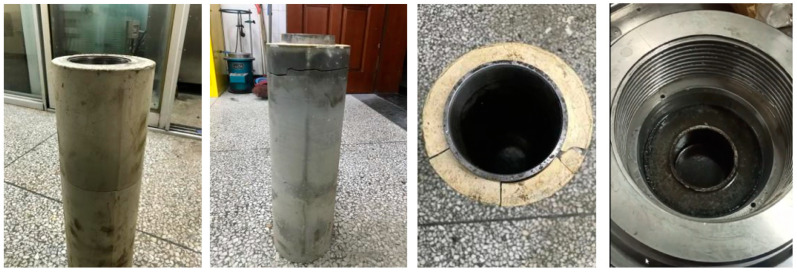
Cracking of cement annulus with blank formulation under 15 MPa cyclic pressure.

**Figure 13:**
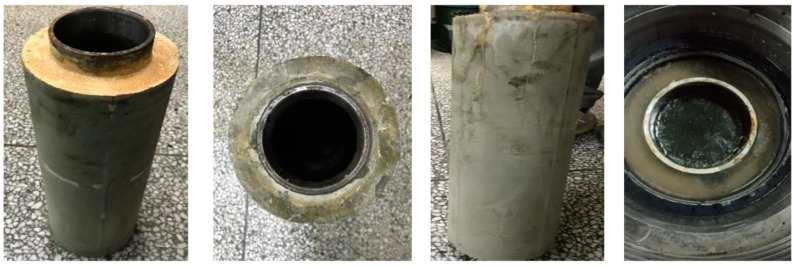
Integrity of cement annulus with toughened formulation under 30 MPa cyclic pressure.

The experimental results in Figure 12 and Figure 13 demonstrate that fiber toughening enhances the integrity of cement annular wellbores under 150 °C curing conditions. For the blank formulation without fibers, under a cyclic alternating pressure of 15 MPa, the cement annulus developed through cracks, severely compromising the sealing performance of the wellbore. At a higher cyclic pressure of 30 MPa, the failure of the cement annulus became even more evident. In contrast, for the fiber-toughened resilient cement slurry, under the same curing conditions and pressure, the cement annulus maintained excellent integrity without any visible cracks or significant damage. The bonding quality remained superior, meeting the requirements for long-term sealing performance.

Additionally, as shown in Table 6, the fiber-toughened cement slurry exhibited stable compressive sealing ability and gas tightness under multiple cyclic alternating pressure tests. The integrity and bonding performance of the cement annulus outperformed the blank formulation. This indicates that fiber toughening effectively improves the toughness and compressive strength of the cement annulus, reducing the risk of crack initiation and propagation and extending the service life of the cement annular wellbore. Therefore, fiber toughening plays a critical role in improving the sealing integrity of cement slurry systems under high-temperature and high-pressure wellbore conditions.

### 3.3. Comprehensive Discussion

The findings of this study demonstrate that modified calcium carbonate whiskers, treated with TEOS, significantly enhance the mechanical properties and sealing performance of oil well cement under high-temperature and high-pressure (HTHP) conditions. The results indicate that a 5% inclusion of modified whisker fibers improved compressive strength by 24.5% and flexural strength by 43.3%, which is consistent with the improvements observed in previous studies. Zhao et al. [11] reported similar enhancements in cement properties through the addition of sulfonate-modified latex powder (SISBR). Their study showed that the inclusion of 2.4% SISBR improved compressive strength to 41.5 MPa and flexural strength to 7.24 MPa. These results are comparable to the mechanical improvements achieved with whiskers in this study. Furthermore, Zhai et al. [12] demonstrated that the use of 15% surface-modified rubber powder in cement enhanced the 28-day compressive strength to 42.4 MPa, which reinforces the role of fiber reinforcement in improving cement durability.

One of the key advantages of whiskers, as demonstrated in this study, is their small diameter, uniform cross-section, and exceptional mechanical strength, which enhance the overall performance of the cement. While traditional short fibers often suffer from agglomeration issues, leading to degraded slurry rheology, whiskers provide better dispersion and integration into the cement matrix, especially when modified with TEOS. This improvement in interfacial bonding is supported by the infrared and X-ray diffraction analyses, confirming that the whiskers are effectively modified to improve their compatibility with the cement. These findings align with the work of Lv et al. [25], who highlighted the benefits of surface-modified whiskers in cement-based materials.

The practical implications of these results are significant for oil and gas cementing operations, particularly in deep or high-pressure wells where traditional cement systems often fail. The enhanced toughness and sealing properties of the modified cement slurry could improve the integrity and longevity of wellbores, reducing the risk of failure during operations such as perforation and fracturing. This approach presents a more reliable and cost-effective solution for cementing in challenging environments.

However, several factors could influence the performance of the modified cement slurry in real-world applications. The compatibility of modified whiskers with other common additives, such as fluid loss control agents and retarders, needs to be carefully evaluated. While the modified slurry showed stable rheological properties and excellent thickening behavior under simulated downhole conditions, its long-term stability in actual wellbore environments, including exposure to fluctuating temperatures, pressures, and aggressive well fluids, requires further research. Studies like Ahmed’s [16] on perlite particles demonstrate the importance of investigating the chemical resistance and durability of modified cement slurries over extended periods.

In summary, the use of modified calcium carbonate whiskers to enhance cement performance offers a promising solution to improve the durability and sealing integrity of oil well cement under extreme conditions. By comparing these results with existing research, it is clear that modified whiskers contribute to stronger, tougher, and more reliable cement slurries. Future studies should explore the long-term performance and scalability of these modified systems to ensure their effectiveness in a wider range of wellbore conditions.

## 4. Conclusions

The study demonstrates that modified calcium carbonate whiskers, treated with tet TEOS, enhance the mechanical properties and sealing integrity of oil well cement under high-temperature and high-pressure (HTHP) conditions. The inclusion of 5% modified whisker fibers results in a 24.5% improvement in compressive strength and a 43.3% increase in flexural strength, supporting the hypothesis that such modifications improve cement slurry performance. Additionally, the fiber-toughened cement slurry exhibits strong sealing performance, maintaining gas tightness and mechanical integrity under cyclic alternating pressures at 150 °C. These findings have significant implications for cementing applications in deep or high-pressure wells, where traditional cement systems often fail due to brittleness and cracking under dynamic loads. The improved toughness and durability of the modified cement slurry offer a practical solution for enhancing wellbore integrity and ensuring long-term zonal isolation in challenging operational conditions.

Further investigation is needed to assess the long-term stability of modified whiskers in actual wellbore environments. It is important to explore the compatibility of modified whiskers with other cement additives and evaluate the slurry’s performance under fluctuating temperature and pressure conditions. These factors are essential for optimizing the formulation and broadening its applicability. The modified whisker-enhanced cement slurry offers an effective approach to improving cementing materials, with the potential for application in complex wellbore environments.

## Figures and Tables

**Table 1:** EDS elemental composition of modified calcium carbonate whiskers.

Element	Wt/%	Atomic/%
C	3.42	7.84
O	18.79	32.32
Si	5.91	5.80
S	27.35	23.47
Ca	44.53	30.57
Total	100	100

**Table 2:** EDS elemental composition of unmodified calcium carbonate whiskers.

Element	Wt/%	Atomic/%
O	30.27	49.6
S	29.27	23.94
Ca	40.46	26.46
Total	100	100

**Table 3:** Mechanical properties of cement stone with different dosages of whisker fibers.

Dosage of Modified Whisker Fibers/%	48 h Compressive Strength/MPa	Compressive Strength Improvement Rate/%	48 h Flexural Strength/MPa	Flexural Strength Improvement Rate/%
0	28.5 ± 1.57	—	6.0 ± 1.21	—
3	31.4 ± 1.33	10.2	7.2 ± 0.88	20.0
5	35.5 ± 1.72	24.5	8.6 ± 1.33	43.3
7	36.4 ± 2.12	27.7	8.9 ± 1.27	48.3

**Table 4:** Properties of cement slurry with modified whisker fibers.

Formula	Density (g/cm^3^)	Fluidity (cm)	Fluid Loss (mL)
Pure Cement Slurry	1.91	23	48
Unmodified Whisker Fiber Cement Slurry	1.90	22	40
Modified Whisker Fiber Cement Slurry	1.90	22	38

**Table 5:** Mechanical properties of cement stone with modified whisker fibers.

Formula	48 h Compressive Strength/MPa	48 h Flexural Strength/MPa	Flexural Strength Increase Rate/%
Pure Cement Slurry	28.5 ± 1.57	6.0 ± 1.21	—
Unmodified Whisker Fiber Cement Slurry	30.5 ± 1.55	7.0 ± 0.86	16.7
Modified Whisker Fiber Cement Slurry	35.5 ± 1.72	8.6 ± 1.33	43.3

**Table 6:** Evaluation of cement annulus integrity under cyclic pressure.

Formulation	Pressure Difference (MPa)	Number of Cycles	Test Pressure (MPa)	Gas Sealing Status	Cement Status
Blank Slurry	15	0	1	No channeling	Intact
	15	10	1	Slight channeling	Intact
	15	20	1	Channeling	Crack
Toughened Cement Slurry	15	0	1	No channeling	Intact
	15	10	1	No channeling	Intact
	15	20	1	No channeling	Intact
	30	10	1	No channeling	Intact
	30	20	1	Slight channeling	Intact

## Data Availability

The original contributions presented in this study are included in the article. Further inquiries can be directed to the corresponding author.
